# Trends of key surveillance performance indicators of acute flaccid paralysis: a descriptive analysis, Uganda, 2015–2020

**DOI:** 10.1186/s12889-022-14077-w

**Published:** 2022-09-07

**Authors:** Bob Omoda Amodan, Annet Kisakye, Patricia Thiwe Okumu, Sherry Rita Ahirirwe, Daniel Kadobera, Alfred Driwale, Alex Riolexus Ario

**Affiliations:** 1 Uganda Public Health Fellowship Program, Kampala, Uganda; 2 Ministry of Health Uganda, Kampala, Uganda; 3 World Health Organization, Country Office, Kampala, Uganda

**Keywords:** AFP, Eradication, Polio, Surveillance, Uganda

## Abstract

**Background:**

Polio is disease caused by poliovirus which can in turn cause irreversible paralytic disease, presenting as Acute Flaccid Paralysis (AFP). A sensitive AFP surveillance system, in which all reported AFP cases are evaluated, first to determine if they are true AFP cases or not, is key for tracking polio eradication. True AFP cases are then later categorized as polio AFP or non-polio AFP (NPAFP) cases. Sensitivity is defined by meeting an annual NPAFP rate/100,000 population < 15 years of ≥ 4/100,000, and an annual stool adequacy (SA) rate of ≥ 80%. We describe Uganda’s AFP surveillance performance between 2015–2020, based on the WHO-recommended indicators, including; NPAFP and stool adequacy rate.

**Methods:**

We performed a descriptive analysis of national AFP surveillance data, 2015–2020 obtained from ministry of health. We evaluated proportion of reported AFP cases that were true AFP, and changes in NPAFP and stool adequacy (SA) rate over the study period. We evaluated the trends in achieving the targeted NPAFP and SA rates from 2015–2020. We used QGIS to illustrate patterns in NPAFP and SA rates across districts and subregions.

**Results:**

Among 3,605 AFP cases reported and investigated countrywide from 2015–2020, 3,475 (96%) were true AFP cases. All the true AFP cases were non-polio related. District reporting was near-complete (97–100% each year). Overall, the mean NPAFP rate declined from 3.1/100,000 in 2015 to 2.1/100,000 in 2020. Less than 40% of districts met the NPAFP target rate in all years. The proportion of districts achieving the NPAFP target rate of ≥ 4/100,000 significantly declined from 35% in 2015 to 20% in 2020. The mean annual SA rate nationally was 88% from 2015–2020. Only 66% of districts achieved the SA target rate of ≥ 80% in the study period. The proportion of districts with SA rate ≥ 80% significantly increased from 68 to 80% between 2015 and 2020.

**Conclusion:**

Most districts reported AFP cases. However, there was a decline in the NPAFP rate from 2015–2020 and few districts achieved the target rate. The suboptimal AFP surveillance system performance leaves the country at risk of missing ongoing poliovirus transmission. We recommend health worker training on active AFP searches, intensified supportive supervision, increase the number of environmental surveillance sentinel sites to boost AFP surveillance in the country, and periodic review meetings with districts to assess AFP surveillance performance.

## Background

Poliomyelitis, also known as polio is a disease caused by poliovirus. Most people infected with poliovirus do not have symptoms, but they can still pass the virus in stool and, for a shorter time, in saliva [1, 2]. About a quarter of people infected experience mild, temporary symptoms such as fever, headache, malaise, nausea, vomiting, and sore throat. In addition, polio can cause lifelong paralysis or death [3]. On average, one in every 200 poliovirus infections present with irreversible paralytic disease, known as Acute Flaccid Paralysis (AFP). Polio is also associated with 5–10% mortality [4]. Between 1988 to 2019, the number of polio cases has reduced from an estimated 350,000 to about 175 [5]. Polio may result from natural infection with wild polioviruses (WPV), or from circulating vaccine-derived poliovirus (cVDPV). cVDPV occurs as a result of virus mutations and spreads in areas with low polio immunization coverage [6]. Poliomyelitis caused by either wild poliovirus or vaccine-derived poliovirus (VDPV), as well as vaccine-associated paralytic poliomyelitis (VAPP) are all targeted for eradication [7]. WPV is a human non-enveloped RNA enterovirus classified as in risk 2 category. Wild polioviruses have three immunologically distinct serotypes (serotypes 1, 2, and 3). Serotypes 2 and 3 were declared globally eradicated by WHO in September 2015 and October 2019, respectively [8]. However, Serotype 1 still circulates and is targeted for eradication [9].

The Global Polio Eradication Initiative (GPEI) Polio Endgame Strategy 2022–2026 targets global eradication worldwide by 2026 [10]. The paths towards eradication of polio infections include, among others, improving detection and responding through sensitive surveillance [10].

A sensitive acute flaccid paralysis (AFP) surveillance system is central to the overall polio eradication initiative [11]. WHO recommends minimum standards for AFP surveillance in a nationwide, case-based syndromic manner with laboratory confirmation of poliovirus from stool specimens. Any AFP cases identified are reported and investigated using both active and passive surveillance and community-based detection methods [3]. All children < 15 years of age presenting with sudden onset of floppy paralysis or muscle weakness affecting limbs, or any person of any age with paralytic illness of with poliomyelitis suspected by a clinician are categorised as AFP cases [12]. AFP cases identified and reported during surveillance can be polio-related or non-polio related. Non-polio related AFP (or Non-polio AFP) is a type of AFP caused by other conditions other than poliovirus such as Guillain-Barré syndrome, transverse myelitis, encephalitis, traumatic neuritis, and other non-polio enteroviruses, among others [13]; these are determined clinically or using laboratory testing.

Uganda Ministry of Health (MoH) adopted and contextualised some of the WHO recommended AFP surveillance performance indicator targets, including, annual NPAFP rate of ≥ 4 per 100,000 children under 15 years of age; and ≥ 80% stool adequacy rate [12]. The NPAFP rate is an indicator of surveillance sensitivity, measured as number of non-polio AFP cases < 15 years / 100,000 persons < 15 years of age. This implies that achieving the target NPAFP rate indicates sufficiently sensitive surveillance to detect WPV/cVDPV cases if poliovirus is circulating. Stool adequacy rate is the proportion of AFP cases with two stool samples of 8–10 g collected ≥ 24 h apart < 14 days after onset of paralysis and arriving at the laboratory in good condition (“Good condition” means that upon arrival: There is ice or a temperature indicator (showing < 8 °C) in the container, with no sign of leakage or desiccations and with proper documentations) [12, 14]. Stool specimen adequacy rate is used to evaluate the investigation timeliness and the quality of the reverse cold chain mechanism employed for poliovirus isolation. Achieving target stool adequacy percentage indicates ability to detect poliovirus among AFP [12].

This assessment describes Uganda’s AFP surveillance performance from 2015 to 2020 using the two key AFP performance indicators of NPAFP rate and stool adequacy rate. This study also explored the oral polio vaccine coverage among AFP cases, and further aimed at generating recommendations to improve key surveillance indicators of acute flaccid paralysis in the country.

## Methods

### Study setting

Uganda is a tropical country located in East Africa, bordered by South Sudan from North, Kenya from the East, Tanzania from the South, Rwanda from the South West, and Democratic Republic of Congo from the West. The projected population of Uganda was 41,583,600 in 2020 with the annual growth rate of 3.3% [15]. As of 2020, Uganda had the projected population of 21,623,472 children under age of 15 years, across all the 135 districts.

### Study design and data source

All reported AFP cases are investigated using a standard case investigation form filled by a health worker. The case investigation form contains sections for clinical, epidemiologic, and laboratory investigations. During investigation of AFP cases, a true AFP case was defined as any child under 15 years of age with acute onset of floppy paralysis within the last 60 days, or a person of any age in who a polio is suspected by a clinician. This information is then recorded in the case-based AFP database. All samples of true AFP cases are taken to the Uganda Virus Research Institute for analysis to determine whether the true AFP was caused by poliovirus (polio related AFP) or non-poliovirus (non-polio related AFP).

We obtained from the MoH aggregated district-level data after secondary analysis of the annual AFP case-based surveillance data. The data included the following performance variables: total cases investigated, true AFP cases, minimum expected number of non-polio AFP cases, non-polio AFP rate, proportion of reported AFP cases investigated within 48 h, proportion of cases whose specimens are arriving at the lab ≤ 3 days, proportion of cases whose specimens are arriving at the lab in good condition, proportion of cases from whose specimens non polio enterovirus (NPENT) was isolated, proportion of cases whose specimens are collected within 14 days of onset, proportion of cases (6 months-15 years) with OPV3 + immunization status, stool adequacy rate, and surveillance index (which is calculated by multiplying the non-polio AFP rate per 100 000 population by the proportion of cases with stool specimens).

### Data analysis

Data merging, manipulation and analysis was done in Excel. We calculated the proportion of districts with non-polio AFP rate of < 1 per 100,000, 1 to < 2 per 100,000, 2 to < 4 per 100,000, and ≥ 4 per 100,000; proportion of districts with OPV3 + immunization status of < 80% and ≥ 80%; proportion of districts with stool adequacy rate of ≥ 80% and percentage of districts that reported at least one case. The analyses were stratified by districts, and calendar year. For spatial analysis, we used QGIS [16] to illustrate differences of non-polio AFP and stool adequacy rate across districts.

## Results

### Acute flaccid paralysis cases and OPV3 + coverage, Uganda, 2015–2020

During the study period, a total of 3,605 AFP cases were reported and investigated throughout the country. Of the reported and investigated AFP cases, 3,475 (96%) were true AFP cases (Table 1). Most of the true AFP cases (85%) had received ≥ 3 doses of oral polio vaccine. Of the true AFP cases, none were classified as polio related, and the NPENT rate was 13% (*n* = 445).

**Table 1:** True acute flaccid paralysis cases reported, Uganda, 2015–2020

Year	Under 15 population	AFP cases investigated	True AFP cases	Cases with OPV3 +	Case in which NPENT isolated
2015	18,461,092	600	570	502	86
2016	19,059,404	713	674	603	66
2017	19,676,228	610	590	434	75
2018	20,310,680	628	606	535	77
2019	20,960,160	595	580	487	88
2020	21,623,472	459	455	401	53
**Total**		**3,605**	**3,475**	**2962**	**445**

### Non-polio AFP rate as a surveillance indicator, Uganda*,* 2015–2020

**Fig. 1:**
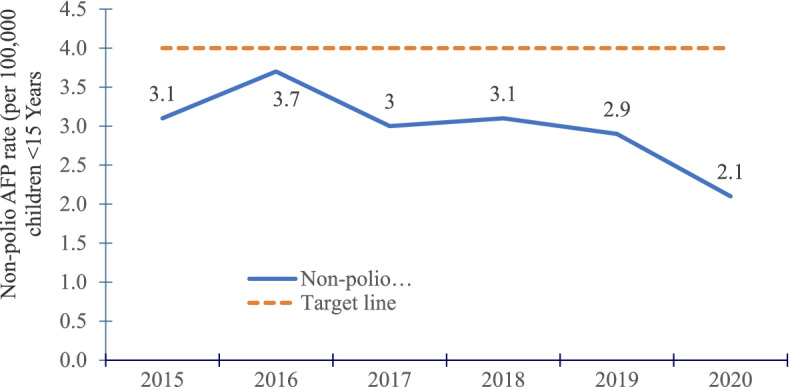
Trends of non-polio AFP performance rate during evaluation of AFP surveillance in Uganda, 2015–2020

**Fig. 2:**
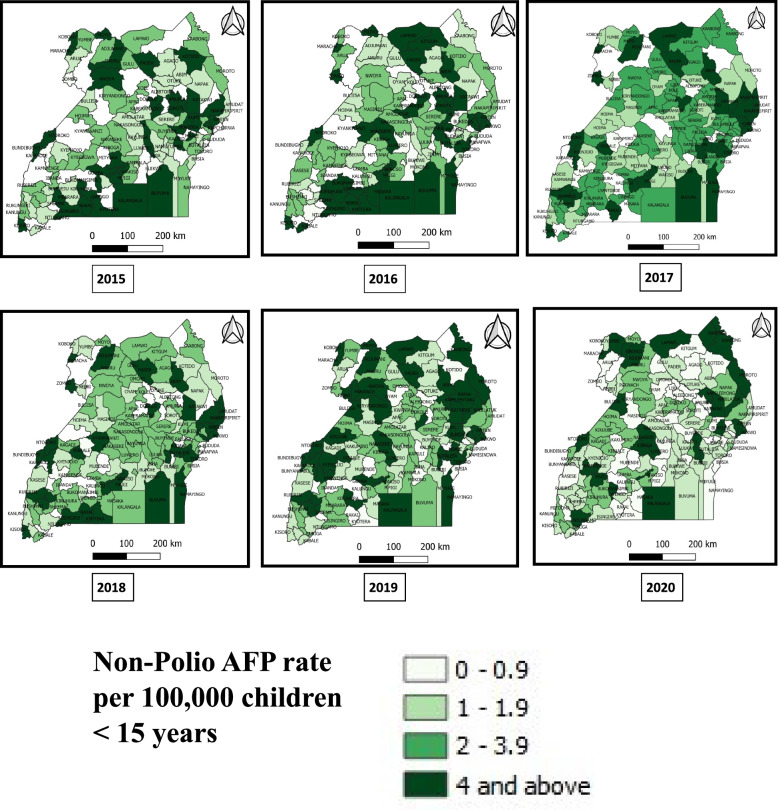
Distribution of Non-polio AFP rate per 100,000 children < 15 years of age by districts, Uganda, 2015–2020

The mean non-polio AFP rate declined from 3.1 per 100,000 in 2015 to 2.1 per 100,000 in 2020 (Fig. 1). Overall, fewer than 40% of districts achieved the non-polio AFP target rate. Although nearly all districts reported, there was an overall decline in proportion of districts with the targeted non-polio AFP rate of ≥ 4 per 100,000 between 2015 and 2020 (Table 2), with only 20% of districts achieving the target in 2020 (Fig. 2).

**Table 2:** District-level non-polio AFP performance in Uganda, 2015–2020

Year	Number of districts*	Non-Polio AFP rate:	Districts with non-Polio AFP rate:				Districts with no report of AFP cases
			** ≥ 4/100000, n (%)**	** ≤ 2 to < 4/100000, n (%)**	** ≤ 1 to < 2/100000, n (%)**	** < 1/100000, n (%)**	
2015	112	3.14	39 (35)	32 (29)	35 (31)	6 (5)	0
2016	112	3.71	40 (36)	37 (33)	25 (22)	10 (9)	1
2017	116	3.01	29 (25)	52 (45)	29 (25)	6 (5)	0
2018	116	3.09	31 (27)	54 (47)	25 (22)	6 (5)	0
2019	128	2.87	49 (38)	37 (29)	31 (31)	11 (9)	0
2020	135	2.10	27 (20)	30 (22)	41 (31)	37 (27)	3

^*^Number of districts: Districts were created by the government once there was need

### Stool adequacy rate as a surveillance indicator, Uganda, 2015–2020

**Fig. 3:**
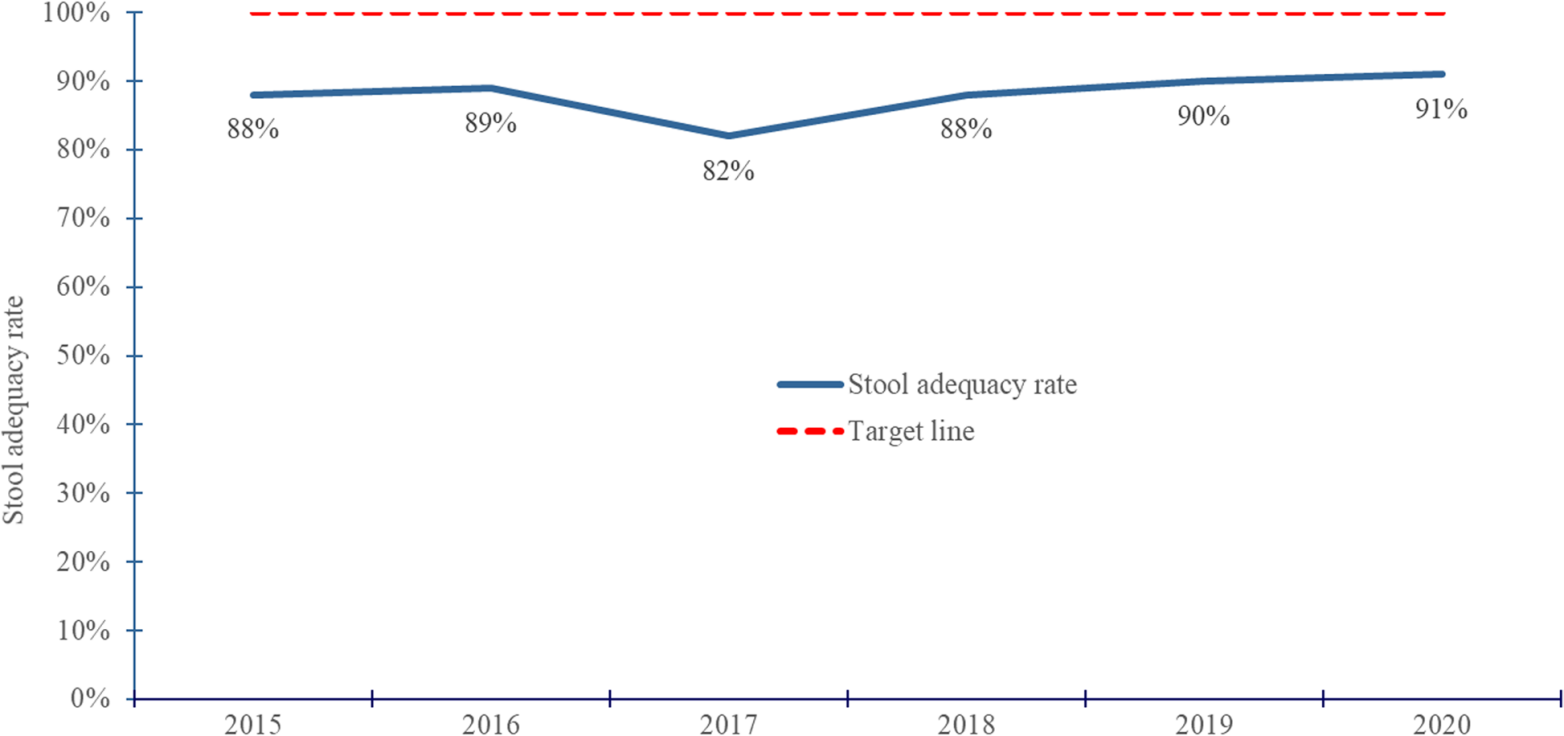
Trend of stool adequacy performance rate during evaluation of AFP surveillance in Uganda, 2015–2020

**Fig. 4:**
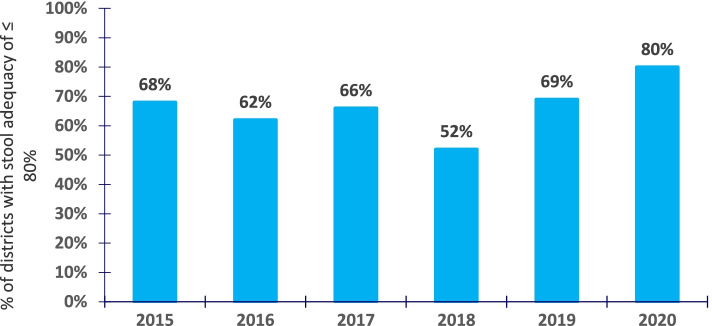
Percentage of districts that achieved the minimum stool adequacy performance rate (of ≥ 80%) in Uganda, 2015–2020

**Fig. 5:**
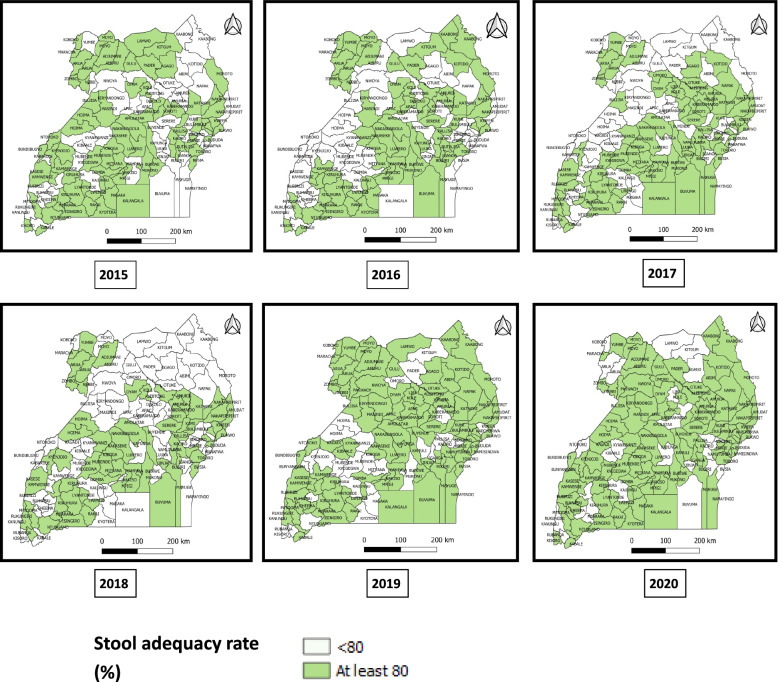
Distribution of stool adequacy rate performance by districts during evaluation of AFP surveillance, Uganda, 2015–2020

Nationally, the stool adequacy rate changed from 88% in 2015 to 91% in 2020 (Fig. 3). Throughout the study period, two-thirds of districts (66%) achieved the SA target rate of ≥ 80% (Fig. 4). The proportion of districts achieving the target SA rate significantly increased from 2015 to 2020 (Table 3) (Fig. 5).

**Table 3:** Stool adequacy performance rates during evaluation of AFP surveillance in Uganda, 2015–2020

Year	Number of districts	Districts attaining stool adequacy rate of ≥ 80%, n (%)	Districts stool adequacy rate of < 80%, n (%)	Mean stool adequacy rate (%)
2015	112	76 (68)	36 (32)	88%
2016	112	69 (62)	43 (38)	89%
2017	116	77 (66)	39 (34)	82%
2018	116	60 (52)	56 (48)	88%
2019	128	88 (69)	40 (31)	90%
2020	135	108 (80)	27 (20)	91%
**Average over the years**		**80 (66)**	**40 (34)**	**88%**

## Discussion

This study aimed at describing the trends AFP surveillance performance between 2015–2020 basing on non-polio AFP, stool adequacy rates and oral polio vaccination coverage among AFP cases reported. Findings indicated that in the study period, most districts reported at least one AFP case. Additionally, most AFP cases investigated in Uganda were true AFP cases, but they were not polio related. Most true AFP cases had received three or more (OPV3 +) doses of oral polio vaccine. The stool adequacy rate was achieved and improved over time from 2015–2020. However, there was a decline in the non-polio AFP rate from 2015–2020, and few districts achieved the target rate.

Most (85%) true AFP cases had received three or more doses of oral polio vaccine. This is similar to study findings in Ethiopia [17], Kenya [18], and Iran [19] which also found that most AFP cases had received three or more doses of oral polio vaccine. While polio vaccination coverage of ≥ 90% yields herd immunity, this assessment indicated that the OPV3 + coverage among AFP cases was lower than required for attainment of herd immunity. However, it is possible that the OPV3 + coverage stated in this study was underestimated or overestimated, as this was self-reported and could have been subject to recall bias [20, 21].

The NPAFP rates of Uganda declined between 2015 and 2020 yet contrasted by many other African countries that have seen an increase in NPAFP rates over similar periods [22]. In addition, Uganda did not meet the set NPAFP target of ≥ 4 per 100,000 children under 15 years. The decline in non-polio AFP rates over the assessment period, and non-attainment of the NPAFP target rates likely indicates decreasing sensitivity of the AFP surveillance system [22]. A study in Kenya recommended improving case detection by strengthening AFP surveillance and active case search in counties and sub counties through training of health workers [18]. The same study, emphasised that subcounty level AFP surveillance data analysis be done to uncover underperformance [18]. It is also possible that district staff lacked a clear understanding of this new non-polio AFP surveillance indicator rate of 4 per 100,000 children < 15 years of age, introduced in 2017. Alternately, they may face insufficient funding or inadequate supervision and feedback [23, 24].

While few districts were ‘silent’ (reported no cases in a year), three districts were silent in 2020 (the highest number of any individual year). ‘Silent’ district is a district, area, or other administrative entity that has not reported a single AFP case for a period ranging from 6 to 12 months or longer. There was poorer performance in meeting the non-polio AFP target rate in 2020 compared to other years: 2020 registered the lowest proportion of districts who met the non-polio AFP target rate of ≥ 4 per 100,000 children under 15 years of age. This might be related to COVID-19-associated interruptions of health service delivery and utilization in the whole country [25, 26]. However, the suboptimal and declining sensitivity of the AFP surveillance system can potentially lead to missed opportunities to detect ongoing transmission of either circulating VDPV or WPV. Environmental surveillance (ES) is a supplementary polio surveillance system, and has become an essential component of the overall polio surveillance program. For example, when the AFP surveillance could not detect any AFP case, the ES findings in the two sentinel sites in Kampala showed presence of an outbreak of poliovirus, as declared on 21 July 2021 in Uganda, following the confirmation of circulating VDPV type 2 linked to the lineage in Sudan [27]. In fact, the nucleotide sequencing of the Uganda’s confirmed circulating VDPV type 2 indicates that the virus had been circulating for over two years in the country [27]. Given this finding, it is even more critical that the surveillance system in Uganda for AFP be strengthened and remain strong.

The proportion of districts with target stool adequacy rate significantly increased between 2015 and 2020, with mean stool adequacy rate in the study period being higher than the recommended target of 80%. This finding is in line with what was found in other countries including Ethiopia [17], some eastern and southern African countries [22], and Liberia [28]. There were sub-regional variations in terms of stool adequacy. This could potentially be due to varied attitudes and skill levels of the health facility staff or district surveillance focal persons in different subregions; however, this remains speculative and needs further investigation.

### Limitations

While the health workers ask for vaccination cards during investigation, they also relied on verbal self-reports as source of vaccination information, which could have led to recall bias, thus either under-estimating or over-estimating the OPV3 + rate.

## Conclusion

Most true AFP cases in Uganda during 2015–2020 had received ≥ 3 doses of oral polio vaccine. Nationally, there was a decline in proportion of districts that achieved the targeted non-polio AFP rate, and an increase in those that achieved the targeted stool adequacy rate between 2015 and 2020. The mean non-polio AFP target rate of 4 per 100,000 children under 15 years was not met by the country, while the average stool adequacy target rate of 80% was met. The worst performance in AFP surveillance was recorded in 2020. The suboptimal AFP surveillance system performance indicated in this study leaves the country at risk of failing to detect any ongoing transmission of either circulating VDPV or WPV.

There is need for the Ministry of Health and it’s relevant partners to train health staff, intensify supportive supervision, increase the number of environmental surveillance sentinel sites to boost AFP surveillance in the country, and hold review and feedback meetings with districts.

## Data Availability

The datasets generated and/or analysed during the current study belong the government of Uganda, Ministry of Health, and therefore not publicly available. However, the data can be availed by the corresponding author with permission from the Ministry of Health, Uganda.

## Abbreviations

AFP: Acute Flaccid Paralysis
GPEI: Global Polio Eradication Initiative
NPAFP: Non-polio AFP
NPENT: Non-polio enteroviruses
OPV3+: Oral polio vaccine (3 or more doses)
SA: Stool Adequacy
VAPP: Vaccine-associated paralytic poliomyelitis
VDPV: Vaccine-derived poliovirus
WHO: World Health Organization
WPV: Wild poliovirus
